# Erratum: Ariani et al., “The Planning Horizon for Movement Sequences”

**DOI:** 10.1523/ENEURO.0500-22.2022

**Published:** 2023-02-06

In the article, “The Planning Horizon for Movement Sequences,” by Giacomo Ariani, Neda Kordjazi, J. Andrew Pruszynski, and Jörn Diedrichsen, which published online on March 22, 2021, an equation appeared incorrectly. On page 4, the second equation in the Data analysis section was originally published as:

$$w^{*}=\frac{-\log(0.01)}{b+1}.$$

This equation should instead appear as:

$$w^{*}=\left(\frac{-\log\left(0.01\right)}{b}\right)+1.$$

Because the equation was used correctly in the analysis code, this error does not affect the conclusions of the article. The online version has been corrected.

